# Supplementary material for: Valorisation, Green Extraction Development, and Metabolomic Analysis of Wild Artichoke By-Product Using Pressurised Liquid Extraction UPLC–HRMS and Multivariate Data Analysis
Source: Molecules. 2022 Oct 22;27(21):7157. doi: 10.3390/molecules27217157 (PMC9656714; doi:10.3390/molecules27217157)
Supplement: Supplementary file 1 [file molecules-27-07157-s001.zip › molecules-1968911-supplementary.pdf]

molecules

Supplementary material

Valorisation, Green Extraction Development, and Metabolomic Analysis of Wild Artichoke By-Product Using Pressurised Liquid Extraction UPLC–HRMS and Multivariate Data Analysis

Stefania Pagliari [1,†], Ciro Cannavacciuolo [1,†], Rita Celano [2], Sonia Carabetta [3], Mariateresa Russo [3], Massimo Labra [1] and Luca Campone [1,*]

[1] Department of Biotechnology and Biosciences, University of Milano-Bicocca, Piazza Della Scienza 2, 20126 Milano, Italy
[2] Department of Pharmacy, University of Salerno, Via Giovanni Paola II 132, Fisciano, 84084 Salerno, Italy
[3] Safety and Sensoromic Laboratory (FoCuSS Lab), Department of Agriculture Science, Food Chemistry, University of Reggio Calabria, Via dell'Università 25, 89124 Reggio Calabria, Italy
* Correspondence: luca.campone@unimib.it
† These authors contributed equally and are co-first authors.

Supplementary material

Table S1. Regression analysis.

Compounds	Linearity (µg mL^{-1})	Calibration Equation	R^2
1-*O*-caffeoylquinic acid	1-15	Y=110765x + 62022	0.9940
chlorogenic acid	1-15	Y=232948x + 136153	0.9661
4-*O*-caffeoylquinic acid	1-15	Y= 61933x + 40499	0.9871
cynarin	1-15	Y= 13893x + 218835	0.9983
scolymoside	1-15	Y= 285215x − 128798	0.9960
luteolin-7-*O*-glucoside	1-15	Y= 100114x + 129507	0.9968
1,4-dicaffeoylquinic acid	1-15	Y= 212364x − 300330	0.9733
1,5-dicaffeoylquinic acid	1-15	Y= 408607x − 65672	0.9949
apigenin-7-*O*-rutinoside	1-15	Y= 200941x − 90663	0.9983
3,5-dicaffeoylquinic acid	1-15	Y= 1036384x − 26373	0.9981
4,5-dicaffeoylquinic acid	1-15	Y= 236028x − 1115	0.9973
luteolin	1-15	Y= 304340x + 138881	0.9966
apigenin	1-15	Y= 516869x − 160184	0.9985
